# Comparing automated surveillance systems for detection of pathogen-related clusters in healthcare settings

**DOI:** 10.1186/s13756-024-01413-5

**Published:** 2024-06-26

**Authors:** Jean Xiang Ying Sim, Susanne Pinto, Maaike S. M. van Mourik

**Affiliations:** 1 Department of Infectious Diseases, Singapore General Hospital, Singapore, Singapore; 2 Department of Infection Prevention & Epidemiology, Singapore General Hospital, Singapore, Singapore; 3 Department of Medical Microbiology and Infection Control, University Medical Center Utrecht, Utrecht, The Netherlands

**Keywords:** Cluster detection, WHONET, SaTScan, Automated surveillance, Pathogen-based detection

## Abstract

**Background:**

Detection of pathogen-related clusters within a hospital is key to early intervention to prevent onward transmission. Various automated surveillance methods for outbreak detection have been implemented in hospital settings. However, direct comparison is difficult due to heterogenicity of data sources and methodologies. In the hospital setting, we assess the performance of three different methods for identifying microbiological clusters when applied to various pathogens with distinct occurrence patterns.

**Methods:**

In this retrospective cohort study we use WHONET-SaTScan, CLAR (CLuster AleRt system) and our currently used percentile-based system (P75) for the means of cluster detection. The three methods are applied to the same data curated from 1st January 2014 to 31st December 2021 from a tertiary care hospital. We show the results for the following case studies: the introduction of a new pathogen with subsequent endemicity, an endemic species, rising levels of an endemic organism, and a sporadically occurring species.

**Results:**

All three cluster detection methods showed congruence only in endemic organisms. However, there was a paucity of alerts from WHONET-SaTScan (*n* = 9) compared to CLAR (*n* = 319) and the P75 system (*n* = 472). WHONET-SaTScan did not pick up smaller variations in baseline numbers of endemic organisms as well as sporadic organisms as compared to CLAR and the P75 system. CLAR and the P75 system revealed congruence in alerts for both endemic and sporadic organisms.

**Conclusions:**

Use of statistically based automated cluster alert systems (such as CLAR and WHONET-Satscan) are comparable to rule-based alert systems only for endemic pathogens. For sporadic pathogens WHONET-SaTScan returned fewer alerts compared to rule-based alert systems. Further work is required regarding clinical relevance, timelines of cluster alerts and implementation.

## Background

Healthcare associated infections cause great mortality and morbidity to patients in the hospital, and adds to rising healthcare costs [1–3]. These infections can be caused by multi-drug resistant micro-organisms that may result in widespread transmission in the hospital setting causing a complex network of infectious disease clusters within wards [2]. Surveillance is key to early identification of possible outbreaks and prevention. Unfortunately, traditional surveillance methods are tedious and time consuming. They often rely on individual clinicians and infection prevention practitioners to raise the alert, resulting in delays in intervention [4]. In recent years, with the shift to an electronic health record (EHR) system, there has been increasing interest in utilizing automated surveillance methods for identifying possible infectious disease clusters, aiming for an earlier and more accurate detection of such clusters [5]. Note that, automated outbreak detection systems (AODS) are not necessarily designed to detect genomically-proven clusters, as this require conformation by, for example, Next Generation Sequencing (NGS) techniques. AODS can however help in early warning, rapid implementation of mitigation, or prioritization of NGS efforts.

Schröder et al. built and implemented an AODS based on routine clinical microbiological examination and reported bacterial pathogens using mathematical algorithms [6, 7]. Another approach is utilizing SaTScan through the WHONET software (freeware developed by the WHO Collaborating Centre for Surveillance of Antimicrobial Resistance at the Brigham and Women’s Hospital in Boston, Massachusetts [8]). This approach enables various analyses using isolate-level microbiology laboratory data. It supports the use of spatiotemporal algorithmic models, such as the space-time continuous uniform model, the space-time discrete Poisson distribution model, and the space-time permutation model [9–11]. In a systematic review of 27 electronic hospital acquired infection (HAI) surveillance systems, it was observed that these systems had higher sensitivity, but lower specificity for the detection of infectious disease clusters compared to manual surveillance performance [4]. Despite the ongoing advancements in this field, numerous unanswered questions remain regarding to this topic. These questions encompass the selection of optimal methods (e.g. a best-fit statistical method) and the level of ease in implementation. The current literature reveals that the heterogeneity of data sources among different studies, methodologies and (cluster) definitions make direct comparison and assessment difficult [12, 13]. 

Moreover, different outbreak patterns can affect the performance of newly created surveillance methods [14]. Pathogen occurrence can be endemic or sporadic, and they may occur irregularly, or as part of an outbreak. These differences need to be taken into account by the automated surveillance methods. This study aims to describe three different methods of identifying microbiological clusters. We do so by applying the systems to the same dataset, obtained from a tertiary hospital. We provide several case studies based on different outbreak patterns to highlight their similarities and differences and discuss their suitability for implementation in the hospital setting.

## Methods

### Setting

This is a single center retrospective cohort study. The University Medical Center Utrecht (UMCU) is a tertiary hospital sited within Utrecht, in the Netherlands. It houses about 1100 beds and is home to specialized units including oncology, neurosurgery, and cardiothoracic surgery. It has a mixed adult surgical and medical intensive care unit (ICU), but also has separate pediatric and neonatal ICUs. The adjacent Central Military Hospital and pediatric oncology center were excluded from this study.

### Baseline dataset curation

Microbiological data were extracted from the laboratory information management system and encompass the period from 1st January 2014 to 31st December 2021. Parameters include the following: specimen number, specimen date, specimen type, isolate number, and antibiotic susceptibility test results. Antibiotic resistance and susceptibility were further qualitatively described as resistant (R), intermediate and from 2021 onwards ‘Susceptible, increased exposure’ (I), and susceptible (S) based on minimum inhibitory concentration (MIC) test results. The laboratory utilises EUCAST references for antibiotic susceptibility [15]. From our EHR database, demographic data were extracted including patient pseudo-ID, sex, age and movement data (ward admission and discharge date) for all patients with a microbiological culture taken.

**Fig. 1:**
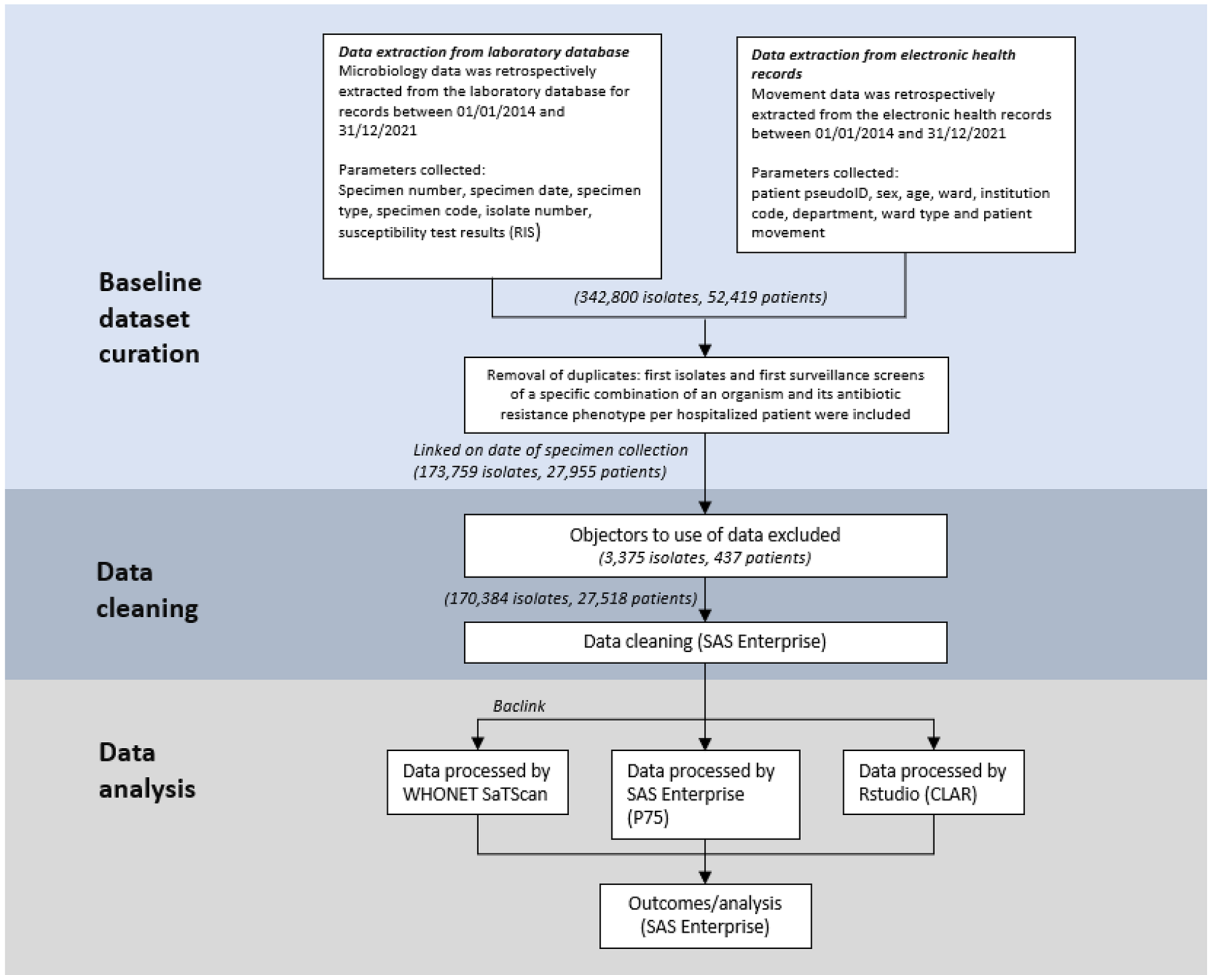
Data flow and processes included in the study

We obtained a waiver of consent from our institutional review board for use of all data, however for purposes of this study persons who registered an objection to use of their medical data were excluded from this study (Fig. 1).

To facilitate for standardized comparison, microorganisms and micro-organism phenotype combinations chosen for this analysis, summarized in Table 1, align with those being surveilled in current standard of care surveillance system. We analyzed the congruency of these specific pathogens of interest between the three surveillance systems described below. We classified all pathogens according to their occurrence in the hospital during the study period (2014–2021) by their endemicity (Table 1). We defined a sporadic organism or group of pathogens as occurring for *≤* 30% of the time intervals and an endemic organism as occurring for > 30% of analyzed time intervals irrespective of absolute numbers. This definition was adopted from the CLAR system to allow for better comparison [6]. We utilize case-studies to illustrate the differences between endemic and sporadic organism with regards to AODS and to facilitate interpretation of data. In addition, we will also describe two further case studies for the following situations [1] introduction of a new pathogen with subsequent low endemicity [2], slow rise in numbers of an endemic organism. The list of pathogens, bug-phenotype combinations, their locations, and their endemicity are described in Table 1.

**Table 1:** Summary of identified pathogens, bug-phenotype combinations, locations and endemicity classified under

*Organism*	*Phenotype*	*Units under surveillance*	*Endemicity*
**Gram-Negative**			
*Acinetobacter spp.*	Meropenem I/R	Hospital-wide	Sporadic
	Quinolone R Aminoglycoside R	Hospital-wide	Sporadic
	All isolates	Adult ICU, NICU	Endemic
*Escherichia coli*	Ciprofloxacin I/R	Hematology	Sporadic
	ESBL	Hospital-wide	Endemic
*Citrobacter freundii*	Ciprofloxacin I/R	Hematology	Endemic
*Enterobacter cloacae*	ESBL	Hospital-wide	Endemic
*Klebsiella pneumoniae*	ESBL	Hospital-wide	Endemic
*Klebsiella oxytoca*	ESBL	Hospital-wide	Endemic
*Serratia marcescens*	All isolates	Adult ICU, NICU	Endemic
*Pseudomonas aeruginosa*	MDR (*≥* R/I to 3 classes of antibiotics)	Hospital-wide	Endemic
	All isolates	Adult ICU, Pediatric ICU	Endemic
	All isolates	NICU	Sporadic
*Stenotrophomonas maltophila*	Cotrimoxazole I/R	Hospital-wide	Sporadic
	All isolates	Adult ICU, Pediatric ICU	Sporadic
	All isolates	NICU	Endemic
Enterobacteriaceae	Meropenem I/R	Hospital-wide	Sporadic
	Quinolone R Aminoglycoside R (excluding *Escherichia coli*)	Hospital-wide	Endemic
*Acinetobacter spp*., *Escherichia coli*, *Enterobacter spp*., *Klebsiella spp*., *Pseudomonas aeruginosa*	Colistin I/R	Hospital-wide	Endemic
**Gram-Positive**			
PRSP	All isolates	Hospital-wide	Sporadic
MRSA	All isolates	Hospital-wide	Endemic
VRE	All isolates	Hospital-wide	Sporadic
*Bacillus spp*.	All isolates	NICU	Sporadic
MSSA	All isolates	Adult ICU, Pediatric ICU, NICU	Endemic
**Fungi**			
*Candida norvegensis*	All isolates	Hematology	Endemic
*Aspergillus fumigatus*	All isolates	Adult ICU	Endemic

Legend:

ESBL: Extended spectrum beta-lactamases

PRSP: Penicillin resistant *Streptococcus pneumoniae*

MRSA: Methicillin-resistant *Staphylococcus aureus*

VRE: Vancomycin-resistant Enterococcus

MSSA: Methicillin-susceptible *Staphylococcus aureus*

ICU: Intensive care unit

*Acinetobacter baumanii*: <08Sep2021 Quinolone I/R, >=08Sep2021 Quinolone R

*Stenotrophomonas maltophilia*: <08Sep2021 CoT I/R, >=08Sep2021 CoT R

*Pseudomonas aeruginosa*: <08Sep2021 Quinolone I/R, >=08Sep2021 Quinolone R

### WHONET-SaTScan

Data collected from the microbiology system was converted to WHONET compatible format through Baclink software. We used the recurrence interval as a measure of statistical significance. We defined the recurrence interval as > 365 days, which indicates that a cluster is supposed to occur by chance only once in more than 365 days. This is in line with previous published studies [16]. The maximum length for which a group of isolates could contribute to a detected cluster was chosen to be 60 days. We tested this parameter at different timepoints (30, 60, and 90 days) with our data set, and we selected a cut off at 60 days, as cluster detection did not improve when longer time intervals were employed [16]. The use of SaTScan via the WHONET software has no significant influence on the results compared to SaTScan software, but the WHONET application is particularly focused on analyzing microbiology laboratory data in hospitals [17]. Although the data was obtained retrospectively, we used WHONET software to simulate a prospective assessment by incrementally adding retrospective data day by day. This allowed us to identify when a cluster was detected, and we present the results from this analysis. A detailed description of the SaTScan system can be found from the SaTScan User Guide [18–20]. 

### CLAR

The CLuster AleRt system (CLAR), developed in-house by Charité University Hospital in Berlin, utilizes data from the previous 15 months to calculate a baseline for organisms of interest [6]. This baseline is then compared to data from the preceding 14 days. The CLAR system employs six different statistical algorithms. When a threshold is crossed for any of the deployed six methods, an alert will be raised. Statistical methods utilized include normal distribution prediction intervals (PI-NV), Poisson distribution (PI-POI), score prediction intervals (PI-SCORE), early aberration reporting system (EARS), negative binominal CUSUMs (NBC), and Farrington algorithm. A detailed description of the CLAR system can be found in Schröder et al. 2020 [6]. 

### P75 system

The current system being utilized in our hospital summarizes the monthly count of a specific microorganism (and micro-organism-phenotype combinations and/or units under surveillance). The threshold for alert is based off the 75th percentile of monthly counts for the preceding year and is manually redetermined each year. Operationally, IP practitioners review the active monthly tally on a weekly basis. A monthly meeting is conducted with microbiologists to discuss the next steps taken for these alerts.

### Data analysis

Microbiological culture results were linked to the ward of sample collection; if a patient was transferred on the day of sampling the culture was attributed to the initial ward. Only first isolates and first surveillance screens of a specific combination of an organism and its antibiotic resistance phenotype per hospitalized patient were included (Fig. 1). Note that it is also possible to select only the antibiotic resistance phenotype and not a specific pathogen (Table 1) for both CLAR and P75 systems, for example for meropenem resistant Enterobacteriaceae. The same input data was used for the comparison of the three outbreak detection methods. The minimum cluster size was defined as 2 patients. Alerts that fall within 60 days (for WHONET SaTScan) or two months (CLAR and P75) of a preceding alert were attributed to initial alert and results are presented in grouped two monthly intervals. The maximum length a group of isolates that could contribute to a detected cluster was set at 60 days or two months depending on the cluster detection system. As the CLAR and P75 system utilizes preceding data for baseline and threshold calculation, only alerts from 1 May 2015 till 31 December 2021 were used for the comparison. General data cleaning and P75 analysis were performed using SAS Enterprise version 9.4. Data was analysed with WHONET-SaTScan version 22.15.15 for WHONET-SaTScan analysis and with R version 3.6.1 for CLAR analysis.

## Results

A total of 173,759 unique isolates belonging to 27,955 patients were collected during the study period. 1716 (6.1%) patients had registered their objection status and 437 (1.6%) patients objected to the use of their medical data and were excluded from further analysis (Fig. 1).

**Fig. 2:**
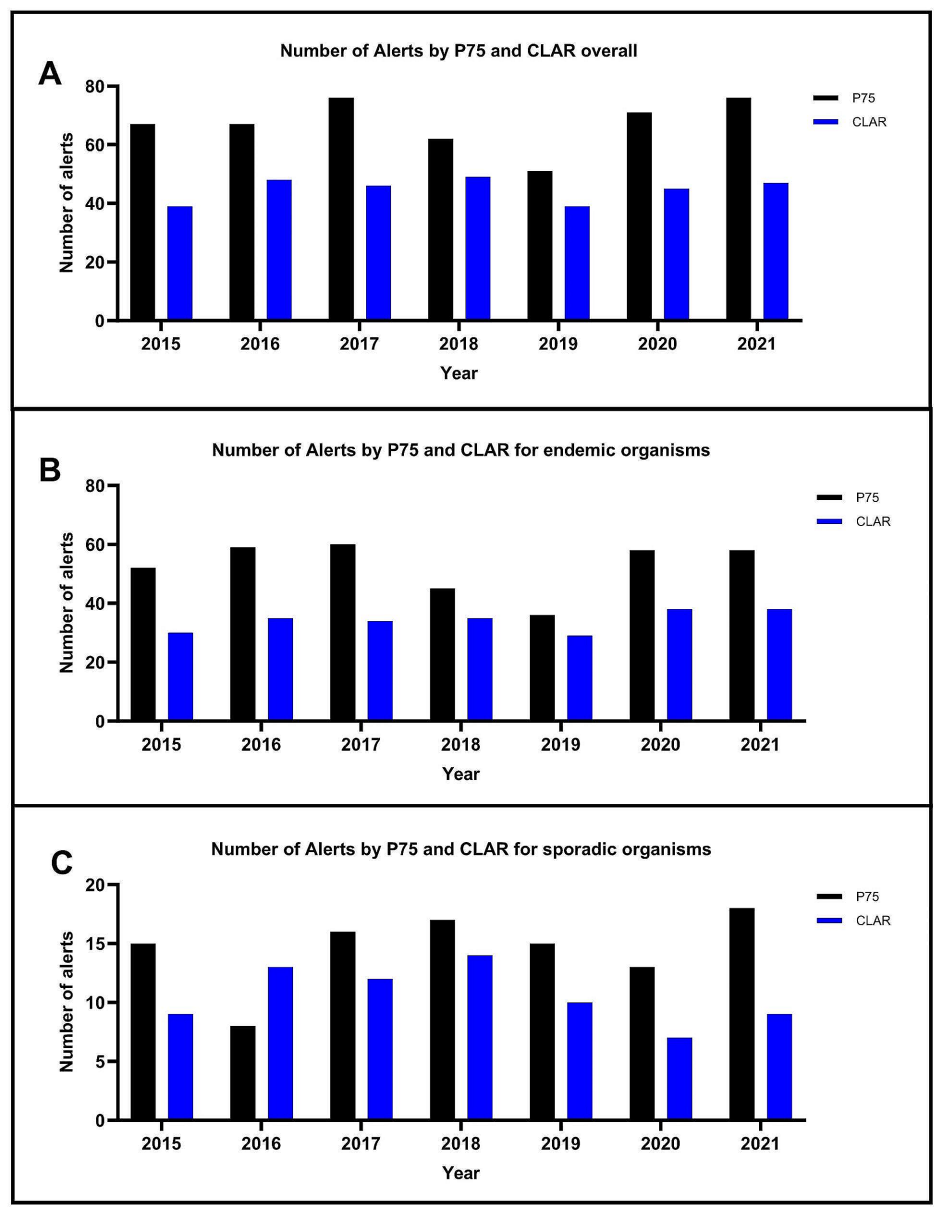
**A**: Total number of cluster alerts generated by the P75 system and by CLAR during the study period. **B**: Cluster alerts generated by the P75 system and by CLAR for endemic organisms during the study period. **C**: Cluster alerts generated by the P75 system and by CLAR for sporadic organisms during the study period

The P75 system generated a total of 472 alerts during the study period of 1 April 2015 to 31 December 2021. During the same period, CLAR identified 319 alerts (Fig. 2) of which 234 alerts were congruent with the P75 system. For endemic organisms, P75 detected a total of 370 alerts and CLAR 244 alerts, of these 183 of the alerts were congruent. For sporadic organisms, P75 detected 102 alerts compared with 75 alerts for CLAR, with 51 congruent alerts.

Given the paucity of alerts from WHONET-SaTScan they were not represented in Fig. 2 but described separately. WHONET-SaTScan identified nine significant clusters (Table 2). Most clusters were also picked up by both the P75 and CLAR system

**Table 2:** Cluster alerts generated by WHONET-SaTScan. The last two columns indicate whether this cluster was also detected by the P75 and/ or CLAR system (Y) or not (N). N.A. means that the specific combination of microorganism and location was not studied with these systems (see also Table 1)

	*Microorganism*	*Location*	*Cluster start date*	*Cluster length (days)*	*Cluster size*	*Detected by P75 system*	*Detected by CLAR system*
1	*Serratia marcescens*	NICU	21/2/2016	43	12	Y	Y
2	*Acinetobacter baumanii*	NICU	1/4/2017	1	2	Y	Y
3	*Escherichia coli*	NICU	24/5/2017	1	3	N.A	N.A
4	*Candida norvegensis*	Hematology ward	5/1/2017	54	11	Y	Y
5	*Serratia marcescens*	NICU	16/8/2017	7	5	Y	Y
6	*Staphylococcus aureus*	NICU	17/4/2019	9	3	Y	N
7	*Citrobacter freundii*	General ward	6/8/2019	1	2	N.A	N.A
8	*Serratia marcescens*	NICU	8/1/2020	42	9	Y	Y
9	*Serratia marcescens*	NICU	20/5/2020	14	6	Y	Y

Legend:

N.A: not applicable

### Endemic organisms

#### Case study 1: Endemic organism (occurring more than 30% of the study time period)

**Fig. 3:**
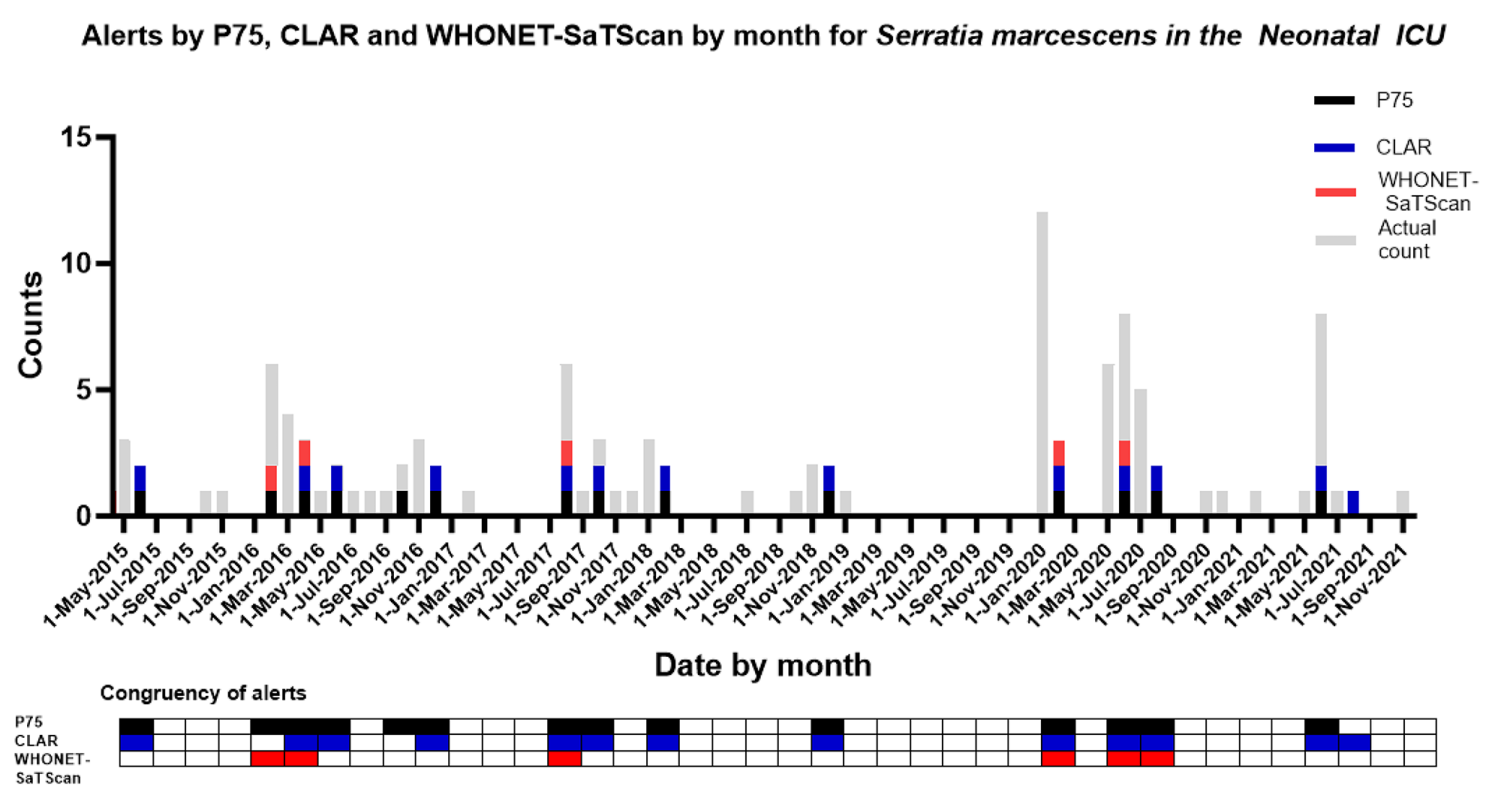
Alerts by P75, CLAR and WHONET-SaTScan cluster detection systems for *Serratia marcescens* in the Neonatal ICU. The (height of the) grey bars indicate the number of positive test cultures per month for a specific pathogen with a specific antibiotic resistance phenotype. The black, blue and red bars and checked boxes indicate the presence of an alert per two months for P75, CLAR and WHONET-SaTScan respectively

In this case study, WHONET-SaTScan generated four clusters during the study period. CLAR and the P75 system generated 13 and 14 alerts, respectively, corresponding to increases in the number of unique patients with *Serratia marcescens* in NICU (Fig. 3).

#### Case study 2: Introduction of a new organism with subsequent endemicity

**Fig. 4:**
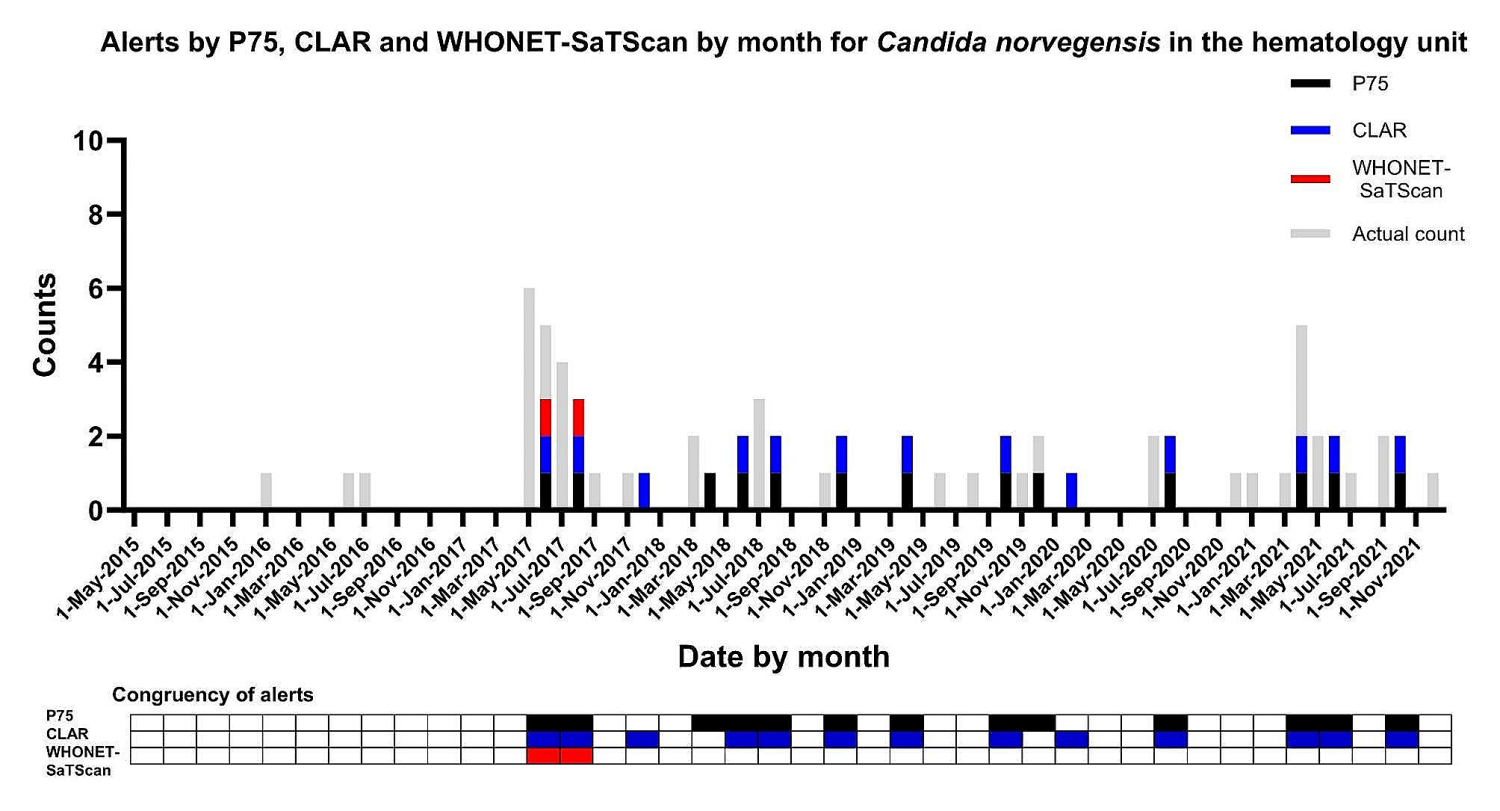
Alerts by P75, CLAR and WHONET-SaTScan cluster detection systems for *Candida norvegensis* isolates in the hematology unit. The (height of the) grey bars indicate the number of positive test cultures per month for a specific pathogen with a specific antibiotic resistance phenotype. The black, blue and red bars and checked boxes indicate the presence of an alert per two months for P75, CLAR and WHONET-SaTScan respectively

*Candida norvegensis* has been a pathogen of interest at the institution within the hematology wards after an initial introduction in 2017. Subsequently, this microorganism transitioned into an endemic species, with occasional increases in the number of cases. There appears to be overall congruence between the P75 and CLAR systems in terms of identifying the overall number of pathogen-related clusters with *Candida norvegensis* (Fig. 4). Both CLAR and the P75 system were able to pick up elevation in numbers of patients with *Candida norvegensis* isolates. WHONET-SaTScan in contrast returned an alert only for the initial introduction of *Candida norvegensis*, but did not detect spikes in endemicity thereafter (Fig. 4).

#### Case study 3: Rising numbers of an endemic organism

**Fig. 5:**
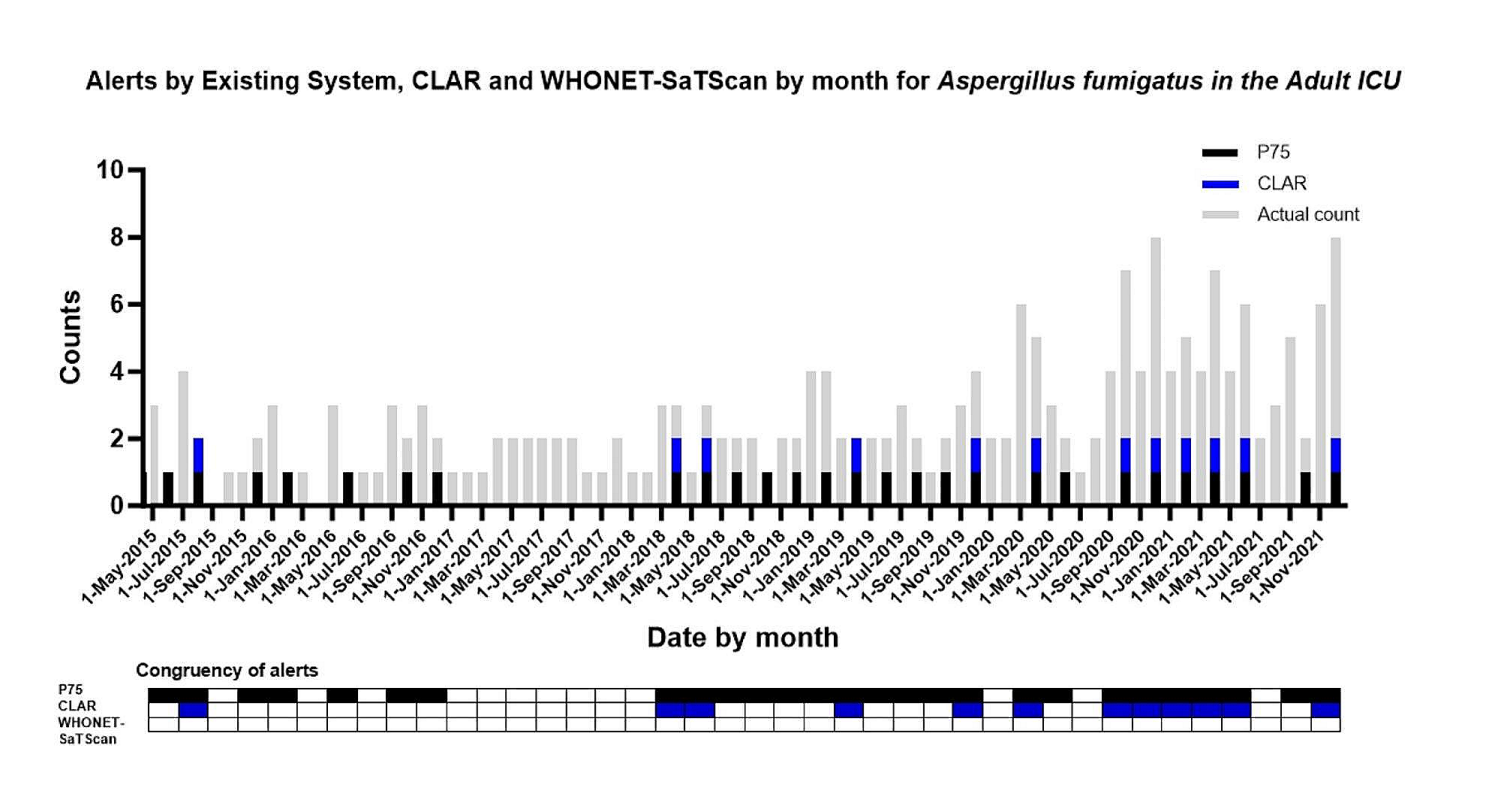
Alerts by P75 system, CLAR and WHONET-SaTScan cluster detection systems for *Aspergillus fumigatus* in the Adult ICU. The (height of the) grey bars indicate the number of positive test cultures per month for a specific pathogen with a specific antibiotic resistance phenotype. The black, blue and red bars and checked boxes indicate the presence of an alert per two months for P75, CLAR and WHONET-SaTScan respectively

*Aspergillus fumigatus* in the adult ICU have been slowly rising in numbers of cases over the years, this was consistently picked up by the rule based P75 system and to another extent the CLAR system, as both utilize previous data to calculate the baseline threshold. However, this trend went unnoticed by the WHONET-SaTScan (Fig. 5), likely because the observed slow increase in cases did not reach a level of statistical significance required by the algorithm for detection.

### Sporadic organism

#### Case study 4: Sporadic occurrence of an organism (occurring less than 30% of the study time period)

**Fig. 6:**
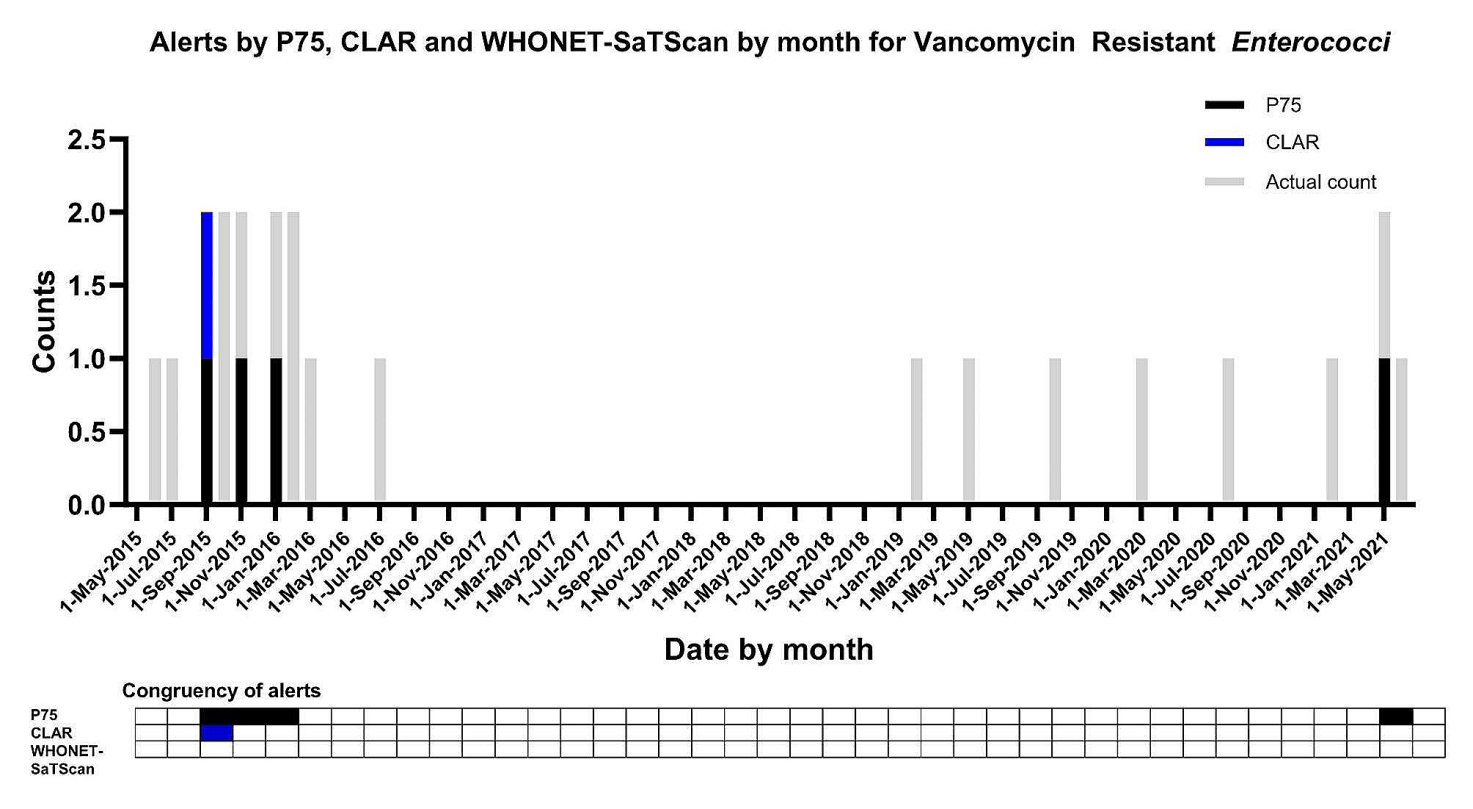
Number of unique patients for VRE and alerts generated by p75, CLAR and WHONET-SaTScan system. The (height of the) grey bars indicate the number of positive test cultures per month for a specific pathogen with a specific antibiotic resistance phenotype. The black, blue and red bars and checked boxes indicate the presence of an alert per two months for P75, CLAR and WHONET-SaTScan respectively

Of the nine clusters identified by WHONET-SaTScan, none of the organisms were deemed sporadic (Tables 1 and 2). Vancomycin Resistant *Enterococci* (VRE) is a sporadic pathogen in our institution. Since 2016 this species is on a declining trend with incidental increases in numbers (less than one case per month). There was one alert of significance identified by the CLAR system. The rule-based P75 system, that utilizes a cut off of 2 patients, returned the most (namely four) alerts. WHONET-SaTScan did not return any alert for VRE (Fig. 6).

## Discussion

We aimed to describe three different AODS algorithms for the detection of pathogen-related clusters and describe their utility. We show the performances of the systems across four different case studies: the introduction of a new pathogen of interest with subsequent low-level endemicity, an endemic organism, slow rise in numbers of an endemic organism, and sporadic occurrence of an organism, and compared their outcomes.

Strikingly, we found a paucity of alerts from the WHONET-SaTScan software as compared to CLAR and the P75 system. However, the clusters that were picked up by WHONET-SaTScan seemed to be worth investigating. This may help reduce the overall number of alerts and allow clinicians to focus on the statistically relevant cluster alerts and reduce user fatigue. For sporadic organisms WHONET-SaTScan did not generate any alerts. There was congruency seen for all three systems for endemic pathogens. However, WHONET-SaTScan did not detect an increase in cases when this happened gradually, as in the case study of *Aspergillus fumigatus* in the adult ICU, while this was constantly alerted by both the CLAR and the P75 systems. Of note, this slow rise in *Aspergillus fumigatus* was detected in the adult ICU since the start of the COVID-19 pandemic, postulated reasons could include COVID-19 associated pulmonary aspergillosis or heightened detection by clinicians due to increased awareness brought on by the pandemic.

Our study is the first to compare the performance of three different outbreak detection methods across four distinct pathogen occurrence case studies for both endemic and sporadic organisms. Our findings align with previous research, indicating that both the CLAR and the WHONET-SaTScan system can detect relevant clusters [16, 21]. The CLAR system can even detect clusters for sporadic pathogens [6], because of its high sensitivity. The trade-off of this increased sensitivity is lower specificity. It is probable that a large portion of increases in numbers, that may trigger an alarm in the P75 and CLAR system, are random events, rather than true outbreaks. In contrast, WHONET-SaTScan can return only the significant outbreaks and ignores small clusters, but the risk of missing a true outbreak, or detecting it too late (when the outbreak is already at its peak), is greater. In any case, a manual verification of the results by infection prevention practitioners remains crucial [22]. The choice of which system to implement should be guided by the specific goals and priorities of the healthcare facility. Therefore, the next step should incorporate an assessment of clinical relevance of the alerts, which can then help to define a best fit system for use by the institution.

In the process of implementing any AODS, it is evident that data pre-processing is considered by institutions before adoption of any system. Both the CLAR and the P75 system require curated data and can only identify clusters of micro-organism-phenotype combinations that are pre-specified. Furthermore, the P75 method requires manual yearly threshold updates, this may not always happen due to resource constraints. In contrast, WHONET-SaTScan has the advantage of analyzing extensive datasets and detecting emerging pathogens within the hospital ecosystem. For CLAR and P75 unfortunately this is not the case, as data needs to be curated prior to being run by the AODS, hence if a novel organism is not specified in the pre-processing phase – this will be missed by both systems. However, due to its comprehensive dataset analysis, WHONET-SaTScan required longer run-times compared to the other two systems but required less curated input data. In addition, it is recognized that in each approach there is the risk that (recent) outbreaks influence future thresholds as the background incidence increases. Finally, it may be considered to improve detection systems by adding a generic rule for new (and hence rare) micro-organism-phenotype combinations based on a simple rule of two or more cases in a 30-day period.

While this study provided valuable insights for infection prevention practitioners who would like to use automated systems for cluster detection, or evaluate their current system, there are some limitations to this study. The analyzed dataset excluded individuals who objected to the use of their medical data. Since the number of such objections was low, this did not significantly impact the final outcomes but may do so if this number continues to rise. A second limitation of this study is that WHONET-SaTScan analyzed the data on a day-by-day basis, while the P75 and CLAR systems both conducted retrospective analysis on the entire dataset. However, it is worth noting that in real-time implementation that the P75 and CLAR systems can be configured to run daily analyses. As this was a retrospective study, time to identification was not studied but should be included in future studies as this would impact upon infection prevention processes whereby timely intervention is key. Denominator information was not incorporated in the analyses, and this could potentially affect cluster analysis with fluctuations in overall patient numbers, this lack of denominator data is current practice in many systems. Differences in endemic and sporadic organisms may need to be factored into the AODS methods, as sporadic species can potentially lower the threshold and increase the number of false positive alerts should there be long periods without occurrences. Likewise, clusters among endemic microorganisms can be missed when the numbers do not exceed the calculated or stipulated thresholds. Data output across all three systems exhibited heterogeneity. To facilitate a fair comparison, we made assumptions regarding maximum cluster length and minimum cluster size. These assumptions may differ among institutions, and our results might not be generalizable to those institutions. Therefore, the assumptions should be tested prior to adoption of each system for every institution. Lastly, with regards to implementation of WHONET SaTScan, although the freeware has improved usability over the original SaTScan, it is less adaptable. Therefore, exploration of the dataset with the original SaTScan package may be important to consider for future studies.

Future studies may improve these methods by including groups of wards that are closely related as epidemiological units and account for difference in underlying number of patient days over time. As this is a retrospective cohort study, real-time assessment of each system will need to be performed to better understand implementation difficulties, technical and data storage issues, and relevance to infection prevention practitioners [23]. In addition, simulation studies may help to investigate the performance of the automated cluster outbreak systems as early warning systems. NGS can serve as a valuable tool to determine clonality within the clusters detected by AODS, and may serve as a gold standard for the comparison of such systems in the future. Integrating NGS data into cluster analysis can enhance the accuracy and reliability of cluster detection, offering insights into the genetic relatedness of microbial isolates within detected clusters other than species-based definitions. For example, transmission of a resistance element (e.g., on a plasmid) may occur in multiple species, and such a transmission event would not be identified using species-based definitions. Moreover, AODS cannot differentiate between hospital acquired infections and community acquired infections, therefore patients within a potential cluster alerted by an AODS can be unrelated [22]. AODS can also help prioritize isolates for NGS testing especially working within real world limitations such as availability, costs, and turnaround time. As such, the choice for a cluster alert system should be tailored to the specific needs of an individual institution. Instead of entirely replacing an existing system, a possible approach is to introduce an additional system as a complementary solution, addressing any shortcomings of the existing one.

## Conclusion

Use of statistically based automated cluster alert systems such as CLAR and WHONET-SaTScan are comparable to rule-based alert systems only for endemic pathogens. For sporadic pathogens, WHONET-SaTScan may result in fewer alerts compared to rule-based alert systems. Further work is required regarding clinical relevance, granularity of cluster alerts and implementation.

## Data Availability

The meta data of our project can be found in the public data repository DataverseNL. Maaike S.M. van Mourik; Susanne Pinto; Jean Xiang Ying Sim, 2024, “Metadata for: Comparing Automated Surveillance Systems for Detection of Pathogen-Related Clusters in Healthcare Settings”, 10.34894/XO0LYD, DataverseNL.

## References

[1] Jarvis WR (1996). Selected aspects of the socioeconomic impact of nosocomial infections: morbidity, mortality, cost, and prevention. Infect Control Hosp Epidemiol.

[2] McFee RB (2009). Nosocomial or hospital-acquired infections: an overview. Dis Mon.

[3] Gidey K, Gidey MT, Hailu BY, Gebreamlak ZB, Niriayo YL (2023). Clinical and economic burden of healthcare-associated infections: a prospective cohort study. PLoS ONE.

[4] de Bruin JS, Seeling W, Schuh C (2014). Data use and effectiveness in electronic surveillance of healthcare associated infections in the 21st century: a systematic review. J Am Med Inf Assoc.

[5] Tinoco A, Evans RS, Staes CJ, Lloyd JF, Rothschild JM, Haug PJ (2011). Comparison of computerized surveillance and manual chart review for adverse events. J Am Med Inf Assoc.

[6] Schröder C, Peña Diaz LA, Rohde AM, Piening B, Aghdassi SJS, Pilarski G (2020). Lean back and wait for the alarm? Testing an automated alarm system for nosocomial outbreaks to provide support for infection control professionals. PLoS ONE.

[7] Aghdassi SJS, Kohlmorgen B, Schröder C, Peña Diaz LA, Thoma N, Rohde AM (2021). Implementation of an automated cluster alert system into the routine work of infection control and hospital epidemiology: experiences from a tertiary care university hospital. BMC Infect Dis.

[8] Stelling J, Yih WK, Galas M, Kulldorff M, Pichel M, Terragno R (2010). Automated use of WHONET and SaTScan to detect outbreaks of Shigella spp. using antimicrobial resistance phenotypes. Epidemiol Infect.

[9] Natale A, Stelling J, Meledandri M, Messenger LA, D’Ancona F. Use of WHONET-SaTScan system for simulated real-time detection of antimicrobial resistance clusters in a hospital in Italy, 2012 to 2014. Euro Surveill. 2017;22(11). 10.2807/1560-7917.ES.2017.22.11.30484 PMC5356424 28333615

[10] Stachel A, Pinto G, Stelling J, Fulmer Y, Shopsin B, Inglima K, Phillips M (2017). Implementation and evaluation of an automated surveillance system to detect hospital outbreak. Am J Infect Control.

[11] Baker MA, Yokoe DS, Stelling J, Kleinman K, Kaganov RE, Letourneau AR (2020). Automated outbreak detection of hospital-associated pathogens: value to infection prevention programs. Infect Control Hosp Epidemiol.

[12] Leclère B, Buckeridge DL, Boëlle PY, Astagneau P, Lepelletier D (2017). Automated detection of hospital outbreaks: a systematic review of methods. PLoS ONE.

[13] Verberk JDM, Aghdassi SJS, Abbas M, Nauclér P, Gubbels S, Maldonado N (2022). Automated surveillance systems for healthcare-associated infections: results from a European survey and experiences from real-life utilization. J Hosp Infect.

[14] Yeng PK, Woldaregay AZ, Solvoll T, Hartvigsen G (2020). Cluster detection mechanisms for Syndromic Surveillance Systems: systematic review and Framework Development. JMIR Public Health Surveill.

[15] EUCAST, Clinical breakpoints and dosing of antibiotics. https://www.eucast.org/clinical_breakpoints. Accessed on 22nd April 2024.

[16] Huang SS, Yokoe DS, Stelling J, Placzek H, Kulldorff M, Kleinman K (2010). Automated detection of infectious disease outbreaks in hospitals: a retrospective cohort study. PLoS Med.

[17] Lefebvre A, Bertrand X, Vanhems P, Lucet JC, Chavanet P, Astruc K (2015). Detection of temporal clusters of Healthcare-Associated infections or colonizations with Pseudomonas aeruginosa in two hospitals: comparison of SaTScan and WHONET Software packages. PLoS ONE.

[18] Kulldorff M. SaTScanTM user guide. Boston; 2006.

[19] Kulldorff M, Heffernan R, Hartman J, Assunçao R, Mostashari F (2005). A space–time permutation scan statistic for disease outbreak detection. PLoS Med.

[20] Kulldorff M (1997). A spatial scan statistic. Commun Statistics-Theory Methods.

[21] Vlek AL, Cooper BS, Kypraios T, Cox A, Edgeworth JD, Auguet OT (2013). Clustering of antimicrobial resistance outbreaks across bacterial species in the intensive care unit. Clin Infect Dis.

[22] Weber A, Neffe L, Diaz LAP, Thoma N, Aghdassi SJS, Denkel LA (2023). Analysis of transmission-related third-generation cephalosporin-resistant Enterobacterales by electronic data mining and core genome multi-locus sequence typing. J Hosp Infect.

[23] van Mourik MSM, van Rooden SM, Abbas M, Aspevall O, Astagneau P, Bonten MJM (2021). PRAISE: providing a roadmap for automated infection surveillance in Europe. Clin Microbiol Infect.

